# Effects of dietary menthol-rich bioactive lipid compounds on zootechnical traits, blood variables and gastrointestinal function in growing sheep

**DOI:** 10.1186/s40104-019-0398-6

**Published:** 2019-12-02

**Authors:** Amlan K. Patra, Sebastian Geiger, Katharina T. Schrapers, Hannah-Sophie Braun, Heidrun Gehlen, Alexander Starke, Robert Pieper, Adam Cieslak, Malgorzata Szumacher-Strabel, Jörg R. Aschenbach

**Affiliations:** 1 Institute of Veterinary Physiology, Freie Universität Berlin, Oertzenweg 19b, 14163 Berlin, Germany; 2 Department of Animal Nutrition, West Bengal University of Animal and Fishery Sciences, 37 K. B. Sarani, Kolkata, 700037 India; 3 PerformaNat GmbH, Hohentwielsteig 6, 14163 Berlin, Germany; 4 Equine Clinic: Surgery and Radiology, Freie Universität Berlin, Oertzenweg 19b, 14163 Berlin, Germany; 5 Clinic for Ruminants and Swine, Faculty of Veterinary Medicine, University of Leipzig, An den Tierkliniken 11, 04103 Leipzig, Germany; 6 Institute of Animal Nutrition, Freie Universität Berlin, Königin-Luise-Strasse 49, 14195 Berlin, Germany; 7 Department of Animal Nutrition, Poznan University of Life Sciences, Wolynska 33, 60-637 Poznan, Poland

**Keywords:** Electrophysiology, Essential oil, Feed intake, Growth performance, Nutrient uptakes

## Abstract

**Background:**

The present study aimed at investigating the influence of 90% menthol-containing plant bioactive lipid compounds (PBLC, essential oils) on growth performance, blood haematological and biochemical profile, and nutrient absorption in sheep. Twenty-four growing Suffolk sheep were allotted into three dietary treatments: Control (without PBLC), lower dose of PBLC (PBLC-L; 80 mg/d) and higher dose of PBLC (PBLC-H; 160 mg/d). Sheep in all groups were fed meadow hay ad libitum plus 600 g/d of concentrate pellets for 28 d.

**Results:**

Average daily gain was not affected by treatment. Feeding of PBLC increased hay and total feed intake per kg body weight (*P* < 0.05). Counts of total leucocytes, lymphocytes and monocytes were not different among treatments. However, neutrophil count decreased (*P* < 0.05) in PBLC-H with a similar trend in PBLC-L (*P* < 0.10). Concentrations of glucose, bilirubin, triglycerides, cholesterol, urea and magnesium in serum were not different among sheep fed different doses of PBLC. However, serum calcium concentration tended to increase in PBLC-H (*P* < 0.10) and serum concentrations of aspartate & asparagine (*P* < 0.01) and glutamate & glutamine (*P* < 0.05) increased linearly with increasing PBLC dose. In ruminal epithelia isolated from the rumen after killing, baseline conductance (*G*_t_; *P* < 0.05) and short-circuit current (*I*_sc_; *P* < 0.01) increased in both PBLC groups. Ruminal uptakes of glucose and methionine in the presence of Na^+^ were not affected by the dietary PBLC supplementation. In the absence of Na^+^, however, glucose and methionine uptakes increased (*P* < 0.05) in PBLC-H. In the jejunum, *I*_sc_ tended to increase in PBLC-H (*P* < 0.10), but baseline *G*_t_ was not affected. Intestinal uptakes of glucose and methionine were not influenced by PBLC in the presence or absence of Na^+^.

**Conclusion:**

The results suggest that menthol-rich PBLC increase feed intake, and passive ion and nutrient transport, the latter specifically in the rumen. They also increased serum concentrations of urea precursor amino acids and tended to increase serum calcium concentrations. Future studies will have to show whether some of these findings might be commonly linked to a stimulation of transient receptor potential (TRP) channels in the gastrointestinal tract.

## Background

Antibiotics have been used as feed additives in livestock and poultry shortly after their discovery from 1940’s [1]. The widespread use of antibiotics as growth promoters has posed a threat due to the development of antibiotic-resistant pathogenic bacteria, which has led to bans or restricted use of antibiotics in several countries [2**]**. This restriction of antibiotics impacts on feed efficiency and growth performance of animals and causes increased incidences of intestinal diseases [1]. Therefore, several alternatives to antibiotic growth promoters including probiotics, prebiotics, enzymes, antimicrobial peptides and plant bioactive compounds have been extensively explored [3–5]. Plant bioactive agents have several beneficial properties including antimicrobial, antioxidant, immune-modulating and pharmacological activities, and thus have been evaluated as natural feed additives in farm animals [3,6,7]. They include flavonoids, tannins, saponins and plant bioactive lipid compounds (PBLC). The latter include primarily the commonly known “essential oils”, which is a misleading term as “essential oils” are not biologically essential and many of them (including menthol) are solid at room temperature, i.e., not an oil. Natural PBLC seem sustainable options that have a history of safe use since ancient times [8]. In ruminants, some PBLC have shown promise to modulate ruminal fermentation favourably, resulting in improved production performance, health status, welfare and environmental advantages [6,9,10].

We recently reviewed the beneficial effects of PBLC on gastrointestinal barrier and transport function, as well as on regulatory pathways involved in these processes [11]. To our knowledge, menthol has not been tested previously for such effects but some data is available from other PBLC. Regarding barrier function, for example, oregano oil fed to pigs upregulated occludin and zonula occludens-1 mRNA expression in the jejunum [12] and olive oil bioactive compounds increased the mRNA levels of E-cadherin-1, occludin and zonula occludens-1 in ileal mucosa of lipopolysaccharide-challenged pigs [13]. Regarding nutrient uptake from the gastrointestinal tract, cinnamaldehyde upregulated the jejunal gene expression of the sodium/glucose co-transporter *SGLT-1* and the facilitated glucose transporter *GLUT-2* [14], and capsicum oleoresin upregulated the mRNA expression of many genes involved in cell membrane channels and transporters in the ileum of pigs [15]. However, a direct proof for the functional relevance of these molecular changes has rarely been provided.

At variance, certain PBLC such as menthol have been shown to directly stimulate the function of bovine transient receptor potential (TRP) channels, which are involved in cation transport across gastrointestinal epithelia [16, 17]. There are also reports that flavonoid compounds could functionally impair nutrient transport mechanisms in the gastrointestinal tract; e.g., quercetin-3-O-glucoside, *trans*-resveratrol and ε-viniferin hindered glucose transport in porcine intestinal epithelial cells *in vitro* [18, 19].

Proceeding from the known role of menthol as TRP channel agonist in the gastrointestinal tract of ruminants [16, 17], we hypothesized that menthol-rich PBLC could have direct effects on ruminal and intestinal epithelia that may be relevant for nutrient absorption and consequently feed utilization beyond the already described effects on ruminal microbial fermentation [20, 21]. Such effects could also be complementary to other known beneficial activities of menthol-containing PBLC such as antioxidant and immunomodulatory effects [22]. Consequently, this study was designed to investigate the effects of PBLC with menthol as the main compound on feed intake and growth performance in growing sheep with an additional focus on ruminal and intestinal glucose and methionine (Met) absorption, blood cell count, and on serum metabolites with relevance to nutrient and mineral homeostasis.

## Material and methods

### Experimental design, animals and management

Twenty-four growing Suffolk sheep (15 females and 9 males) were purchased from a local farmer. Sheep were group-fed and adapted to a control diet for at least 14 d before allocating them to different diets. At the start of the experiment, body weight (BW) and age of the animals were 32.9 ± 3.44 kg and 121 ± 3.75 d, respectively. The experiment was conducted in two runs with 12 sheep in each run. Sheep were equally allocated into three dietary treatments in each run in a randomized block design based on initial body weight and sex, each treatment containing 5 females and 3 males. The three groups were 1) Control diet (without PBLC), lower dose of PBLC (PBLC-L; 80 mg/d) and higher dose of PBLC (PBLC-H; 160 mg/d). In each run, sheep were kept in four indoor pens with each pen containing three separate feeding stations. Sheep were equipped with electronic transponders on their neck collar that opened the automatic locking gates of one transponder-operated feeding station (Hütter GbR, Marktbergel, Germany). They were trained for 2 to 4 d until they recognized their individually allocated feeders easily. Pens had concrete floor with wood shavings as bedding material. The room was lighted by natural day-light from glass windows along with artificial light from 06:00 to 18:00 h.

### Diet preparation and feeding

During the adjustment period to the automatic feeding system, all sheep were fed the pelleted Control concentrate (400 g/d) and ad libitum meadow hay (without chopping). Thereafter, the experiment started with providing the three different concentrates. The amounts of concentrates were gradually increased: 450 g/d for the first 3 d, 525 g/d for the next 3 d, and 600 g/d thereafter. Hay was provided in the forage storage containers of the feeding stations for ad libitum intake. Ingredients and chemical composition of the three concentrates were identical, except that concentrates of the PBLC-L and PBLC-H groups were added with PBLC at 133.3 and 266.7 mg/kg concentrates (as-fed basis; Table 1). The PBLC contained 900 g/kg menthol together with other minor PBLC components. The PBLC components were added to the concentrates as a commercial premix (OAX17, PerformaNat GmbH, Berlin, Germany) with ground corn as carrier. Ad libitum hay plus 600 g/d pelleted concentrate diets were fed to meet nutrient requirements according to NRC [23]. Water was available at all times from push-button water troughs. The daily dose of PBLC was supplied with the concentrate pellets that were provided in three equal portions of 200 g each at 07:00, 11:00 and 15:00 h. Concentrate mixtures were pelleted below 50 °C to prevent loss of PBLC during pelleting. Concentrate pellets were stored in air-tight bags.

**Table 1:** Ingredient and chemical composition of pelleted concentrates and hay fed to sheep

Item	Concentrate^a^			Hay
	Control	PBLC-L	PBLC-H	
Ingredient composition, g/kg as-fed				
Corn	305	295	285	
Barley	305	305	305	
Soybean meal	348	348	348	
Molasses	30	30	30	
Mineral and vitamin premix^b^	5	5	5	
Salt	2	2	2	
Limestone	5	5	5	
PBLC premix^c^	0	10	20	
Chemical composition				
Dry matter (DM), g/kg as-fed	914	915	912	923
Organic matter, g/kg DM	949	950	948	958
Crude protein, g/kg DM	259	257	259	108
Ether extract, g/kg DM	30.8	25.1	25.8	9.60
Neutral detergent fibre, g/kg DM	125	140	152	641
Acid detergent fibre, g/kg DM	65.4	74.6	70.7	374
Crude fibre, g/kg DM	48.9	41.7	43.0	332
Calcium, g/kg DM	5.31	5.36	5.83	3.11
Phosphorus, g/kg DM	5.09	4.81	4.74	2.08

^a^Control: without PBLC; PBLC-L: lower dose (133.3 mg/kg) of PBLC; and PBLC-H: higher dose (266.7 mg/kg) of PBLC

^b^Mineral and vitamin premix (Spezialfutter Neuruppin Ltd., Neuruppin, Germany), containing per kilogram dry matter: 160 g calcium, 40 g phosphorus, 100 g sodium, 30 g magnesium, 500,000 IU vitamin A, 50,000 IU vitamin D_3_, 500 mg vitamin E (as alpha-tocopherol acetate), 4500 mg zinc (as zinc oxide), 500 mg manganese (as Mn-(II)-oxide), 20 mg cobalt (as Co-(II)-carbonate), 20 mg iodine (as calcium iodate), and 35 mg selenium (as sodium selenite)

^c^PBLC premix (OAX17, PerformaNat GmbH, Berlin, Germany; containing 13.3 g PBLC/kg corn grains)

### Body weight, feed intake and feed sampling

Body weight of sheep was measured at 7-day intervals at 09:30 to 10:00 h. Body weight (BW) and BW changes of the first week were not used to account for potential rumen fill effects due to dietary shifts. As such, initial and final body weight, and average daily gain are presented over the last 21 d of the experiment. Concentrate intake was recorded routinely every day; there were no orts of concentrate. Orts of hay were collected before concentrate feeding in a bag and pooled per animal and per week. Hay intake was measured at weekly interval subtracting the amount of hay orts from the amount of hay offered in a week. Intake is reported on a dry matter basis over the last 21 d of the feeding trial, again, considering the first week as adaptation to the diets. Hay samples were obtained weekly and pooled together per run. Concentrate and orts samples were also obtained weekly and pooled per run and per treatment. Samples of hay, orts and concentrates were kept at − 20 °C until analysis.

### Blood cell count and biochemical profile

Blood samples were collected from the jugular vein using two 9-mL tubes (Vacuette® Greiner Bio-One GmbH, Kremsmüster, Austria) between 09:30 to 10:00 h, i.e. 2.5 h after the morning feeding of concentrates, on d 0 (1 d before actual start of the experiment) and on d 27. One vacutainer tube with clot activator was used for harvesting of serum and another tube with K3-EDTA was used for haematological profile. Vacutainers for serum were allowed to clot for 30 min at room temperature and then put on ice. Serum was separated by centrifugation (1,500×*g* at 4 °C for 10 min) within 1 h after blood sampling and immediately stored at − 20 °C until analysis. The serum concentrations of glucose, triglycerides, bilirubin, cholesterol, urea, calcium and magnesium were measured by a Cobas C 311 (Roche Diagnostics Deutschland GmbH, Mannheim, Germany) in the laboratory of the Clinic for Internal Veterinary Medicine of Leipzig University (Leipzig, Germany) with ready-to-use *e* pack reagents (Roche Diagnostics Deutschland GmbH). Haematological profile of blood was analysed within 2–4 h using an automated haematology analyser (CA530-VET, Boule Medical, Stockholm, Sweden). Briefly, the analyser determined cell counts and volumes by electronic impedance. Mean corpuscular volume, mean platelet volume and red cell distribution width were determined from the size distribution curve of erythrocytes or platelets automatically. Haemoglobin concentration was determined photometrically while mean corpuscular haemoglobin concentration and mean corpuscular haemoglobin were calculated using established equations by the in-built software of the analyser. Packed cell volume (PCV) was determined by the microcentrifugation method. The PCV samples were centrifuged at 3,400×*g* for 15 min (Haematokrit 210, Hettich, Tuttlingen, Germany).

### Amino acid analysis in serum

The concentrations of free amino acids in serum samples were determined by chromatographic UPLC analysis [24]. The derivatization procedure followed the instruction of the kit AccQ•Tag reagent (Waters, Milford, MA, USA). Briefly, 50 μL serum sample was transferred to screw neck max recovery vials (Waters, Milford, MA, USA), 20 μL of borate buffer was added to each vial and the solution was briefly vortexed. Following this, 20 μL working internal standard, 90 μL 10 mmol/L hydrochloric acid and 20 μL of 10 mmol/L 6-aminoquinolyl-N-hydroxysuccinimidyl carbamate in acetonitrile were added to the vials and the solution was vortexed again for 10 s before being heated at 55 °C using thermo-block MD-02 N (Major Sciences, Saratoga, CA, USA) for 10 min. The standard solution (Amino Acid Standard H; ThermoScientific, Rockford, IL, USA) of amino acids mixture contained His, Ser, Arg, Gly, Asp, Glu, Thr, Ala, Pro, Lys, Tyr, Val, Ile, Leu, Phe and Met at a concentration of 2.5 mmol/L, whereas concentration of Cys was 1.25 mmol/L. With this method, Gln is analyzed together with Glu, and Asn is analysed together with Asp. DL-2-aminobutyric acid (Sigma-Aldrich; St. Loius, MO, USA) was used as an internal standard. The UPLC system (ACQUITY; Waters, Milford, MA, USA) fitted with photodiode array detector was used for determination of free amino acids. Chromatographic separation was obtained with the AccQ-Tag Ultra C-18 column (2.1 mm × 100 mm; 1.7 μm). The flow rate was 0.6 mL/min and a binary gradient system with two eluents was used for separation, eluent A (consisting of 25 mL concentrated AccQ-Tag Ultra diluted in 500 mL MilliQ water) and eluent B (Amino acids analysis; AccQ-Tag Ultra; Waters). The gradient system was as follows: 0–0.64 min, 99.9% A; 0.64–6.71 min, 90.9% A; 6.71–7.74 min, 78.8% A; 7.74–10.11 min, 40.4% A; 10.11–12 min, 99.9% A. The injection volume was 1 μL, and the column temperature was set at 55 °C. The amino acid peaks in the samples were identified by comparison to the retention times of the amino acid peaks of the standard solution. Thereafter, the concentrations of the amino acids in the samples were calibrated using the peak area of the standard amino acid concentrations (Empower 2 software; Waters).

### Feed analysis

Chemical composition of feed and orts samples was analysed following standard methods [25]. Content of dry matter was determined in a drying cabinet (VDLUFA MB III 3.1), crude ash in a muffle furnace at 550 °C (VDLUFA III 8.1), ether extract by the Soxhlet method with hydrolysis (VDLUFA MB III 5.1.1) and crude protein (CP) by incineration method (VDLUFA MB III 4.1.2). Acid detergent fibre expressed exclusive of residual ash (ADF_om_) was determined by using Fibertec™ 8000 (FOSS, Hilleroed, Denmark; method, VDLUFA MB III 6.5.2). Neutral detergent fibre content (comparable to assay with heat-stable amylase and expressed exclusive of residual ash; aNDF_om_) was estimated by near-infrared spectroscopy according to the method VDLUFA MB III 31.2. Dietary calcium and phosphorous contents were determined by inductively coupled optical emission spectroscopy (ICP-OES; DIN EN ISO 11885:2009–09).

### Uptake of glucose and methionine

In the week following the 28-d feeding trial, animals were maintained on their dietary protocol according to their group assignment until captive bolt stunning and killing by exsanguination. In a rotating order among groups, two animals were slaughtered per day; one animal 2 h after the morning feeding and a second animal 2 h after the noon feeding. Preparation and incubation of ruminal and intestinal tissues largely followed previously described protocols [26]. Pieces from the ventral ruminal sac and mid-jejunum were dissected after slaughter and rinsed with NaCl solution (9 g/L; 38 °C). Tissue pieces were transferred to transport solution (Table 2) pre-gassed with carbogen (5% CO_2_ and 95% O_2_) at 38 °C, where epithelia were stripped off the muscular and serosal layers. The stripped epithelia were placed in a Dewar container with fresh transport buffer (carbogen-gassed; 38 °C) and transported to the laboratory within 30 min. Epithelia were mounted in Ussing chambers with an exposed area of 3.14 cm^2^. Total time taken from euthanasia to mounting in Ussing chambers was ~ 45 min. The chambers contained 16 mL incubation solution on the serosal and the mucosal side each (Table 2). The serosal solution was the same for all treatments and contained glucose (10 mmol/L) and Na^+^ (135 mmol/L). The mucosal solutions were all glucose-free, and Na^+^ was either present (135 mmol/L) or completely replaced by *N*-methyl-*D*-glucamine (NMDG^+^). To simulate the rumen conditions *in vivo*, mucosal solutions for ruminal epithelia contained short-chain fatty acids (SCFA) and were titrated to pH 6.4. All solutions were circulated by a gas lift system (5% CO_2_ and 95% O_2_) and maintained at 38 °C with the aid of thermostated water jackets around the solution reservoirs. Two chambers per epithelial tissue (rumen and jejunum) and per each mucosal buffer (Na^+^-containing and Na^+^-free) were used for uptake measurements in each sheep.

**Table 2:** Chemical composition of mucosal and serosal buffers used in the Ussing chamber study

Item	T&S solution	Mucosal solutions, rumen		Mucosal solutions, intestine	
		Na^+^-containing	Na^+^-free	Na^+^-containing	Na^+^-free
Composition, mmol/L					
CaCl_2_	1.8	1.8	1.8	1.8	1.8
MgCl_2_	1.0	0	0	0	0
HEPES, free acid	10.0	10.0^a^	10.0^a^	10.0	10.0
NaCl	70.0	70.0	0.0	70.0	0.0
HCl	0.0	17.0	87.0	2.0	72.0
Na-acetate	0.0	25.0	0.0	0.0	0.0
Acetic acid	0.0	0.0	25.0	0.0	0.0
Na-propionate	0.0	10.0	0.0	0.0	0.0
Propionic acid	0.0	0.0	10.0	0.0	0.0
Na-butyrate	0.0	5.0	0.0	0.0	0.0
Butyric acid	0.0	0.0	5.0	0.0	0.0
Na-gluconate	40.0	0.0	0.0	40.0	0.0
Gluconic acid	0.0	0.0	0.0	0.0	40.0
NMDG free base	5.0	2.0	112	7.0	117
KH_2_PO_4_	0.4	0.4	0.4	0.4	0.4
K_2_HPO_4_	2.4	2.4	2.4	2.4	2.4
NaHCO_3_	25.0	25.0	0.0	25.0	0.0
Cholin- HCO_3_	0.0	0.0	25.0	0.0	25.0
Enrofloxacine	0.0278	0.0278	0.0278	0.0278	0.0278
Mannitol	0	19.0	19.0	9.0	9.0
Glucose	10.0	0	0	0	0
Osmolarity, mOsmol/L	288	288	288	288	288
pH	7.4	6.4	6.4	7.4	7.4

HEPES: 4-(2-hydroxyethyl)-1-piperazine ethanesulfonic acid; NMDG: *N*-methyl-*D*-glucamine; T&S: Transport and serosal

^a^Ruminal mucosal buffer contained MES (2-morpholinoethanesulfonic acid) free acid instead of HEPES free acid

Approximately 10 min after mounting, all epithelia were set to short-circuit mode, i.e. the transepithelial potential difference (*PD*_t_) was clamped to 0 mV by the aid of a computer-controlled voltage-clamp device (Mussler Scientific Instruments, Aachen, Germany). The inverse value of the current required to clamp *PD*_t_ to 0 mV was continuously recorded as short-circuit current (*I*_sc_), representing the energy-dependent charge transfer across the epithelium. Additionally, transepithelial conductance (*G*_t_) as a measure of ion conductivity was measured from the changes in *PD*_t_ after bipolar current pulses (100 μA for 300 ms at 6-s intervals) according to Ohm’s law (*G*_t_ = *δI/δPD*_t_) [26]. All epithelia were equilibrated for about 30 min under short-circuit conditions with Na^+^-containing buffers at the mucosal side until *G*_t_ stabilized. The plateau values of *G*_t_ and *I*_sc_ after equilibration were averaged over 1 min and used for statistical comparison among animals.

After equilibration, uptake measurements were initiated with modifications from previously established protocols [27]. The mucosal solution was discarded and Na^+^-free or Na^+^-containing mucosal solutions were filled up in accordance with chamber assignments for both ruminal and intestinal tissues. Partially radiolabeled [^3^H]-*D*-glucose (74 kBq/16 mL) and [^14^C]-*L*-methionine (37 kBq/16 mL) were added simultaneously to final concentrations of 50 μmol/L (*L*-methionine) and 200 μmol/L (*D*-glucose) to the mucosal solution. Hot samples (100 μL) were taken from the mucosal solution in duplicate at 20 s. After 1 min incubation, the solutions on both sides of the epithelium were drained out and the epithelium was flushed with ice-cold intestinal Na^+^-free solution three times to wash off all extracellularly adhering radioactivity.

The epithelia from the Ussing chambers were quickly transferred on the top of 15 mL Falcon tubes (exposed area, 1.77 cm^2^) prefilled with 5 mL of 0.2 mol/L NaOH and 0.25% SDS solution. The tissues were vortexed for 2 min to lyse cells and the tubes were placed on the rotator for another 30 min. The lysate was collected into 15 mL Falcon tubes and centrifuged at 5,000×*g* and 4 °C for 20 min. Supernatant samples (800 μL) were mixed with 3 mL Aquasafe 300 (Zinsser Analytic GmbH, Eschborn, Germany) for determination of radioactivity using a liquid scintillation counter (LKB Wallace-Perkin-Elmer, Überlingen, Germany). The specific activity (cpm per pmol substrate) were calculated from the radioactivity of the hot samples and used to calibrate the radioactivity of cold samples to uptakes of glucose and methionine. Uptakes were expressed as pmol/(cm^2^ · min).

### Statistical analyses

Intake, growth and nutrient uptake data were analysed using mixed model procedures of SAS [28]. The model included treatment, block, experimental run and sex. If effects of block, sex or experimental run were not significant (*P* > 0.05) and increased *P*-values, they were removed before final analysis. Blood-biochemical variables at 28 d were analysed using the same model but using 0 d data as covariates. Since CP intake could be a factor affecting serum amino acid concentrations, amino acids that were different at *P* < 0.10 during initial analysis were additionally analysed using CP intake as covariate to test if these amino acid concentrations increased due to the CP intake or PBLC effects. Linear and quadratic effects of PBLC doses (0, 80 and 160 mg/d) were assessed using polynomial contrasts. Contrasts between Control (0 mg/d of PBLC) versus both PBLC groups (80 and 160 mg/d of PBLC) were also used to determine the overall effects of PBLC compared with the Control. In addition, least significant difference (LSD) post hoc test was used to elucidate differences between PBLC-L or PBLC-H versus Control. If outliers (> or < median ± 2.5 median absolute deviation) were detected [29], they were removed with a missing observation. Variability in the data was expressed as the pooled SEM, and statistical significance was set at *P* ≤ 0.05, while a trend was considered at 0.05 < *P* ≤ 0.10. Correlation between serum glucose concentrations and glucose uptakes in the rumen or intestine was determined using proc. corr of SAS [28].

## Results

### Growth and nutrient intake

Final BW as well as average daily gain of sheep was similar among treatments (Table 3). Supplementation of PBLC in diets tended to increase hay intake dose-dependently (*P* = 0.090) so that hay intake tended to be higher for PBLC-L and PBLC-H each compared with Control (*P* < 0.10). Because all sheep consumed offered concentrates completely, this also resulted in a trend for linear increase (*P* = 0.091) in total feed intake that was reflected in a trend for higher feed intake when comparing PBLC-L or PBLC-H to Control individually (*P* < 0.10). Crude protein intake also increased linearly (*P* = 0.022) with a trend for increase when comparing Control versus PBLC-L (*P* < 0.10) and a significant increase when comparing Control versus PBLC-H (*P* < 0.05). Daily hay intake and total feed intake normalized per kg BW tended to be greater (*P* < 0.10) in PBLC groups when compared to Control individually and were greater (*P* < 0.05) when comparing both PBLC groups together to Control. Intake of NDF was not different among treatments, though it was numerically greater in the PBLC treatments.

**Table 3:** Effect of two doses of dietary menthol-rich plant bioactive lipid compounds (PBLC) on daily body weight (BW) gain and daily nutrient dry matter intake in sheep

Attribute	Treatment^a,b^			SEM	Contrast^c^		
	Control	PBLC-L	PBLC-H		1	2	3
Initial BW, kg^c^	35.5	34.3	35.9	0.83	0.69	0.18	0.71
Final BW, kg	39.1	38.4	39.7	0.63	0.47	0.43	0.84
Average daily BW gain, g/d^d^	170	195	180	12.8	0.61	0.32	0.34
Hay intake, g/d	660	707^#^	725^#^	25.6	0.090	0.65	0.091
Hay intake, g/(d · kg BW)	17.6	19.1^#^	19.0^#^	0.55	0.091	0.25	0.047
Total feed intake, g/d	1209	1255^#^	1272^#^	25.0	0.094	0.64	0.093
Total feed intake, g/(d · kg BW)	32.4	34.1^#^	33.4^#^	0.51	0.17	0.073	0.041
Crude protein intake, g/d	213	215^#^	225*	3.58	0.022	0.40	0.096
NDF intake, g/d	532	552	578	19.5	0.12	0.77	0.13

NDF: neutral detergent fibre; SEM: standard error of mean

^a^Control: without PBLC; PBLC-L: lower dose (80 mg/d) of PBLC; and PBLC-H: higher dose (160 mg/d) of PBLC

^b^One outlier for average daily gain (g/d) < 97, hay intake (g/d) < 525, total feed intake (g/d) < 1126, and hay intake (g/kg BW) < 15.3 was removed for PBLC-L (*n* = 7) before statistical analyses

^c^Contrast: 1 = linear effect; 2 = quadratic effect; and 3 = Control versus both PBLC groups

^d^Average daily gain and the initial BW to calculate this gain are based on the last 21 d of the study to nullify the effect of rumen fill during the first week that might have occurred due to changes in feed intake by PBLC

^#/^*Mean values differ compared with the control at *P* < 0.10/0.05

### Blood haematological and biochemical profile

Total leucocyte, lymphocyte and monocyte numbers were not different among treatments (Table 4). However, neutrophil counts tended to be lower for PBLC-L versus Control (*P* < 0.10) and were lower for PBLC-H versus Control (*P* < 0.05) or when comparing both PBLC groups versus Control (*P* = 0.031). This resulted in a quadratic decrease in proportion of neutrophils (*P* = 0.019) coupled to a quadratic increase in the proportion of lymphocytes (*P* = 0.020), which were also reflected in decreased proportion of neutrophils and increased proportions of lymphocytes when comparing PBLC-L or PBLC-H individually or jointly to Control (*P* < 0.05). The proportion of monocytes was unaffected by treatments. Various erythrocyte and platelet profiles were analysed, which were not influenced by treatments (Table 4).

**Table 4:** Effect of two doses of dietary menthol-rich PBLC on different haematological values in sheep

Attribute	Treatment^a,b^			SEM	Contrast^c^		
	Control	PBLC-L	PBLC-H		1	2	3
Leucocyte profile							
Total leucocytes, ×10^9^/L	15.2	13.4	14.1	0.72	0.34	0.19	0.12
Lymphocyte, ×10^9^/L	12.0	10.9	12.0	0.060	0.95	0.16	0.41
Lymphocyte, %	79.7	85.0**	83.0*	1.09	0.044	0.020	0.006
Monocyte, ×10^9^/L	0.074	0.068	0.072	0.0038	0.65	0.29	0.34
Monocyte, %	0.48	0.51	0.51	0.026	0.38	0.78	0.39
Neutrophil, ×10^9^/L	2.96	2.26^#^	2.18*	0.277	0.055	0.34	0.031
Neutrophil, %	19.8	14.4**	16.5*	1.08	0.042	0.019	0.005
Erythrocyte profile							
Total erythrocytes, × 10^6^/L	15.0	14.6	14.7	0.46	0.66	0.65	0.54
Haemoglobin, g/dL	12.5	12.2	12.5	0.38	0.94	0.53	0.70
PCV, %	43.7	44.4	44.3	1.41	0.76	0.80	0.69
MCV, fL	23.1	23.9	23.4	0.64	0.79	0.44	0.53
MCH, pg	8.30	8.36	8.40	0.23	0.76	0.96	0.78
MCHC, g/dL	35.8	35.2	36.0	0.70	0.89	0.42	0.70
RDWc, %	28.4	28.3	28.8	0.70	0.67	0.72	0.85
RDWs, fL	24.7	25.0	25.1	0.62	0.66	0.90	0.66
Platelet profile							
Platelets, ×10^6^/L	368	372	389	61.3	0.81	0.93	0.87
MPV, fL	5.15	5.08	5.14	0.11	0.94	0.62	0.75
PCT, %	0.19	0.19	0.20	0.031	0.84	0.81	0.96
PDWc, %	21.6	21.2	21.7	0.55	0.95	0.52	0.79
PDWs, fL	3.84	3.70	3.80	0.16	0.87	0.55	0.66

*PCV* packed cell volume, *MCV* mean corpuscular volume, *MCH* mean corpuscular haemoglobin, *MCHC* mean corpuscular haemoglobin concentration, *RDWc* red cell distribution width (co-efficient of variations), *RDWs* red cell distribution width (standard deviation), *MPV* mean platelet volume, *PCT* plateletcrit, *PDWc* platelet distribution width (co-efficient of variations); and PDWs: platelet distribution width (standard deviation)

^a^Control: without PBLC; PBLC-L: lower dose (133.3 mg/kg) of PBLC; and PBLC-H: higher dose (266.7 mg/kg) of PBLC

^b^One outlier for lymphocyte (%) < 70 in PBLC-L (n = 7) and neutrophil (%) > 29 in PBLC-L (n = 7) and leucocyte number > 22.9 × 10^9^/L in PBLC-H (*n* = 7) was removed before analysis

^c^Contrast: 1 = linear effect; 2 = quadratic effect; and 3 = Control versus both PBLC groups

^#/^*^/^**Mean values differ compared with the control at *P* < 0.10/0.05/0.01

Concentrations of glucose, bilirubin, triglycerides, cholesterol, urea and magnesium in serum were not different among sheep fed different doses of PBLC (Table 5). However, there was a trend for a linear increase in serum calcium concentrations due to PBLC feeding (*P* = 0.089), which was also reflected by a trend (*P* < 0.10) for increased serum calcium values for PBLC-H versus Control .

**Table 5:** Effect of two doses of dietary menthol-rich PBLC on selected serum metabolites in sheep

Attribute	Treatment^a^			SEM	Contrast^b^		
	Control	PBLC-L	PBLC-H		1	2	3
Glucose, mmol/L	4.04	3.95	3.98	0.065	0.49	0.43	0.32
Calcium, mmol/L	2.62	2.62	2.68^#^	0.022	0.089	0.41	0.26
Bilirubin, μmol/L	0.76	0.86	0.81	0.107	0.74	0.57	0.57
Tryglyceride, mmol/L	0.23	0.22	0.24	0.022	0.81	0.63	0.98
Cholesterol, mmol/L	1.60	1.65	1.64	0.044	0.53	0.44	0.41
Magnesium, mmol/L	1.00	1.01	1.01	0.018	0.90	0.99	0.91
Urea, mmol/L	9.38	10.1	9.61	0.382	0.67	0.21	0.32

*SEM* standard error of mean

^a^Control: without PBLC; PBLC-L: lower dose (80 mg/d) of PBLC; and PBLC-H: higher dose (160 mg/d) of PBLC

^b^Contrast: 1 = linear effect; 2 = quadratic effect; and 3 = Control versus both PBLC groups

^#^Mean values differ compared with the control at *P* < 0.10

The concentrations of free amino acids in serum were not changed (*P* > 0.10) except for Asp+Asn, Glu + Gln, Lys and, as a trend, Pro and Gly (Table 6). Concentrations of Asp+Asn (*P* = 0.005) and Glu + Gln (*P* = 0.041) increased linearly with increasing doses of PBLC and both were or tended to be different in comparisons of Control versus PBLC-L (*P* < 0.10 for Asp+Asn; *P* < 0.05 for Glu + Gln) or PBLC-H (*P* < 0.05 for both). The difference between Control versus both PBLC groups was also significant for theses amino acids (*P* = 0.009 and *P* = 0.020, respectively). The PBLC addition to the diet further tended to increase concentrations of Gly quadratically (*P* = 0.058) with a trend for higher concentrations for PBLC-L versus Control (*P* < 0.10) and Lys linearly (*P* = 0.072) with higher concentrations for PBLC-H versus Control (*P* < 0.05). Finally, serum concentrations of Pro tended to be increased when comparing PBLC-L to Control (*P* < 0.10) or when comparing both PBLC groups to Control (*P* = 0.054). When CP intake was used as a covariate, concentrations of Asp+Asn (linear effect, *P* = 0.034; Control versus both PBLC, *P* = 0.041), Glu + Gln (Control versus PBLC, *P* = 0.041) and Gly (quadratic effect, *P* = 0.071) were or tended to be increased by PBLC. However, Pro and Lys concentrations were not altered (*P* > 0.10).

**Table 6:** The effect of menthol-rich PBLC on concentrations (μmol/L) of free amino acids in serum of sheep

Item	Treatment^a^			SEM	Contrast^b^		
	Control	PBLC-L	PBLC-H		1	2	3
Ala	60	61	61	0.6	0.30	0.45	0.20
Val	118	120	118	2.4	0.96	0.50	0.70
Leu	133	137	137	1.8	0.14	0.49	0.11
Ile	133	136	136	1.3	0.19	0.46	0.14
Phe	223	227	228	2.5	0.17	0.63	0.15
Met	154	154	154	0.6	0.75	0.92	0.82
Pro^c^	103	105^#^	105	1.0	0.12	0.32	0.072
Gly^c^	60	66^#^	59	2.5	0.85	0.058	0.41
Ser	82	82	84	1.7	0.42	0.72	0.60
Cys	200	200	201	0.7	0.13	0.39	0.37
Thr	113	115	115	1.2	0.30	0.50	0.22
Tyr	289	298	290	4.3	0.84	0.11	0.32
Asp+Asn^c^	121	125^#^	128*	1.4	0.005	0.79	0.009
Glu + Gln^c^	160	174*	174*	4.5	0.041	0.22	0.020
Lys^c^	147	148	152*	1.5	0.054	0.50	0.17
His	171	171	168	1.9	0.31	0.62	0.52
Arg	345	353	341	11.9	0.81	0.41	0.83
Total	2611	2672	2651	32.0	0.42	0.52	0.68

*SEM* standard error of mean

^a^Control: without PBLC; PBLC-L: lower dose (80 mg/d) of PBLC; and PBLC-H: higher dose (160 mg/d) of PBLC

^b^Contrast: 1 = linear effect; 2 = quadratic effect; and 3 = Control versus both PBLC groups

^c^Since CP intake could be a factor affecting serum amino acid concentrations, CP intake was used as covariate for amino acids those resulted in *P* < 0.10, and the statistical results are provided in the text

^#/^*Mean values differ compared with the control at *P* < 0.10/0.05

### Electrophysiology and uptakes of glucose and methionine in gastrointestinal epithelia

Pre-feeding of PBLC increased *I*_sc_ linearly in ruminal epithelia (*P* < 0.001) and also tended to increase *I*_sc_ linearly in intestinal epithelia (*P* = 0.085; Table 7). *I*_sc_ values were higher in PBLC groups when tested either individually or jointly against Control in ruminal epithelia (*P* < 0.01) and tended to be higher when comparing PBLC-H or both PBLC groups with Control in intestinal epithelia (*P* < 0.10). Baseline *G*_t_ increased linearly with increasing doses of PBLC in the rumen (*P* = 0.013) based on greater values for PBLC-L (*P* < 0.05) or PBLC-H (*P* < 0.01) versus Control; however, *G*_t_ was not altered in the jejunum (Table 7).

**Table 7:** Effect of two doses of dietary menthol-rich PBLC on ruminal and jejunal short-circuit current [*I*_sc_; μEq/(cm^2^ · h)], conductance (*G*_t_; mS/cm^2^) and uptakes [pmol/(cm^2^ · min)] of glucose (*U*_gluc_) and methionine (*U*_met_) in the presence (+ Na^+^) or absence of Na^+^ (− Na^+^)

Attribute	Treatment^a,b^			SEM	Contrast^c^		
	Control	PBLC-L	PBLC-H		1	2	3
Rumen							
*I*_sc_ (+ Na^+^)	10.8	21.8**	20.6**	1.46	< 0.001	0.004	< 0.001
*G*_t_ (+ Na^+^)	3.38	3.62*	4.40**	0.26	0.013	0.41	0.065
*U*_gluc_ (+ Na^+^)	237	251	274	36.6	0.48	0.92	0.57
*U*_gluc_ (− Na^+^)	203	194	255*	17.5	0.053	0.12	0.33
*U*_met_ (+ Na^+^)	56.2	60.8	71.2	11.3	0.36	0.84	0.49
*U*_met_ (− Na^+^)	50.3	45.7	67.9*	5.48	0.036	0.061	0.35
Jejunum							
*I*_sc_ (+ Na^+^)	5.27	9.40	9.91^#^	1.81	0.085	0.43	0.063
*G*_t_ (+ Na^+^)	28.9	26.6	27.7	1.96	0.66	0.49	0.47
*U*_gluc_ (+ Na^+^)	1239	1465	1145	151	0.66	0.16	0.73
*U*_gluc_ (− Na^+^)	842	893	746	127	0.60	0.53	0.89
*U*_met_ (+ Na^+^)	715	817	603	94.0	0.40	0.18	0.97
*U*_met_ (− Na^+^)	376	463	365	55.3	0.89	0.19	0.59

*SEM* standard error of mean

^a^Control: without PBLC; PBLC-L: lower dose (80 mg/d) of PBLC; and PBLC-H: higher dose (160 mg/d) of PBLC

^b^One animal in each treatment was removed due to high conductivity

^c^Contrast: 1 = linear effect; 2 = quadratic effect; and 3 = Control versus both PBLC groups

^#/^*^/^**Mean values differ compared with the control at *P* < 0.10/0.05/0.01

Ruminal uptakes of glucose and methionine were not affected by the dietary PBLC in the presence of Na^+^. In the absence of Na^+^, however, uptake of glucose tended to increase linearly (*P* = 0.053) and methionine uptake increased linearly (*P* = 0.036), which was based on higher uptakes of glucose and methionine in PBLC-H compared with Control (*P* < 0.05). In the jejunum, uptakes of glucose and methionine were not influenced by PBLC in the presence or absence of Na^+^.

**Fig. 1:**
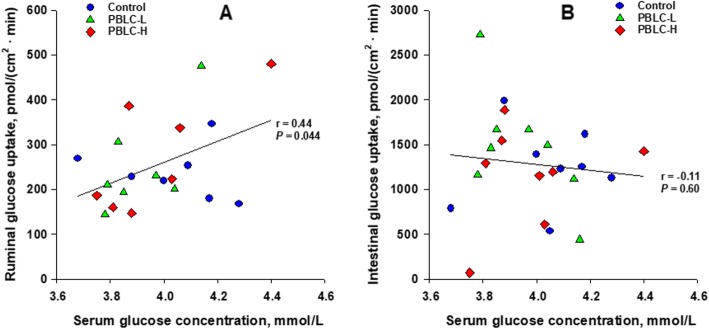
Correlations between serum glucose concentrations and ruminal glucose uptakes (**a**) or intestinal glucose uptakes (**b**) in the presence of Na^+^ in sheep. Control: without PBLC; PBLC-L: lower dose (80 mg/d) of PBLC; and PBLC-H: higher dose (160 mg/d) of PBLC

Across treatments, the absence of Na^+^ tended to decrease the ruminal uptake of glucose [from 250 to 211 pmol/(cm^2^ · min); SE = 17.1; *P* = 0.089], but not of methionine [62.2 versus 51.1 pmol/(cm^2^ · min); SE = 5.33; *P* = 0.23]. Intestinal uptakes of glucose [1,283 versus 827 pmol/(cm^2^ · min); SE = 82.4; *P* < 0.001] and methionine [727 versus 421 pmol/(cm^2^ · min); SE = 46.2; *P* < 0.001] greatly differed in the presence versus absence of Na^+^. Overall, uptakes were about 5 and 10 times greater for glucose and methionine, respectively, in the intestine than in the rumen. A positive correlation (*r* = 0.44; *P* = 0.044) was discovered between ruminal glucose uptakes in the presence of Na^+^ and serum glucose concentrations (Fig. 1). However, no such correlation was noted between intestinal glucose uptakes and serum glucose concentrations.

## Discussion

Hay intake and consequently total feed intake increased due to PBLC addition to the diet. Menthol has characteristic gustatory and olfactory stimulating effects that may affect feed intake and eating behaviour of sheep [30]. In a previous study where crystalline menthol (0, 30, 300 and 3,000 mg/kg diet) was supplemented to steers, overall DM intake was not affected by treatments, but was influenced by an interaction between treatment × day. Steers consumed less feed when supplemented with the highest dose of 3,000 mg/kg diet of menthol from d 5 through 12, but DM intake was similar among treatments after day 13 [31]. In the present study, we wanted to avoid any depression of feed intake and used two doses of PBLC in the lower range of that previous study (80 to 160 mg/d; equivalent to a menthol dose of 57 to 114 mg/kg diet DM). Those low doses appeared to stimulate appetite, resulting in higher hay and total feed intake per unit of BW. Intake of CP increased linearly in parallel, whereas NDF intake was only numerically higher for the PBLC treatments. The latter was attributable to numerically higher concentrations of NDF in the orts samples for the PBLC treatments. We recently identified that the observed stimulation of appetite by low-dose menthol extended beyond the periods when the menthol-rich concentrates are provided, i.e., it was also evident for hay consumption between concentrate feeding hours [32].

Feeding of other PBLC resulted in inconsistent effects on feed intake in different studies. In agreement with our study, clove or cinnamon oil (3.5 and 7 g/d in bulls) [9], rosemary oil (0.5 g/kg concentrate in sheep) [33] and cinnamaldehyde (0.4 to 1.6 g/d in steers) [34] increased feed intake. Some studies reported no effect on feed intake (0.5 and 10 g/d of a mixture of cinnamaldehyde and eugenol, and 0.25 g/d of capsicum oil in lactating Holstein cows [35]; copaiba oil at 0.5 to 1.5 g/kg DM in sheep [36]; 110 mg/d of peppermint oil in sheep [37]); whereas few studies reported lower feed intake (PBLC mixture containing as main components thymol, eugenol, vanillin, and limonene at 1.2 g/d in lactating dairy cows [38]). The variable effects on feed intake in different studies might be due to the fact that type and concentrations of applied PBLC differed strongly between experiments.

The higher feed intake per kg BW was not correlated with higher average daily weight gain in the present study. This might be due to the relatively short duration of the study where small increases in daily weight gain may be difficult to identify. Another possibility could be that PBLC decreased digestibility. Because SCFA concentrations were not altered; however, it is rather unlikely that PBLC impacted on daily BW changes via decreased diet digestibility. Alternatively, menthol has been shown to increase TRP-M (melastatin) channel-mediated brown fat thermogenesis and metabolic rate in humans [39,40]. In lactating dairy cows, dietary addition of peppermint herb (*Mentha piperita*; 50 g/kg diet) with menthol as the main PBLC similarly increased heat production [41]. The increased energy expenditure and heat production during menthol supplementation possibly originates from chemically induced cold sensation because menthol is a well-described agonist of the TRPM8 channel that functions as a cold receptor [42]. This might partly explain the missing effect on body weight gain despite higher feed intakes in menthol-supplemented animals. Similar to our study, no effect on BW gain was observed after addition of crystalline menthol to diets of steers [31] or after supplementing menthol-containing PBLC blends to growing lambs [4] or calves [43].

Supplementation of PBLC at 80–160 mg/d to the diets did not alter blood biochemical profile in the present study, except for a trend for increased serum calcium levels in the PBLC-H group (160 mg/d). In previous studies, changes of blood biochemical profiles due to dietary PBLC were occasionally observed and primarily affected the lipid profile [7, 44, 45]. Serum calcium concentrations were rarely monitored in those studies. We had included the measurement of calcium and magnesium in our protocol since it has been shown recently that menthol and some other PBLC can activate TRP channels in the gastrointestinal tract [16, 17]. These channels play a role in cation absorption with special importance for divalent cations. Accordingly, a recent study had already shown significant increases in serum calcium levels of dairy cows after supplementing a menthol-rich PBLC similar to that of the present study [46]. Although the increase in serum calcium was not significant in the present study, it points into the same direction that menthol-rich PBLC can support calcium homeostasis of ruminants, most likely, by stimulating calcium absorption from the gastrointestinal tract.

Concentrations of selected amino acids in serum were increased by menthol-rich PBLC, which could possibly be due to increased CP intake [47] considering the increased CP intake during PBLC feeding in the present study. However, the selectively increased concentrations of Glu + Gln and Asp+Asn, especially at the higher dose (160 mg/d), were independent (using CP intake as a covariate) of CP intake, which strongly suggests that PBLC was causative for this response. As just elaborated for calcium, menthol has been shown to activate TRP channels in the gastrointestinal tract which, in the rumen, leads to increased NH_4_^+^ absorption [16, 17]. Increased absorption of NH_4_^+^ requires increased Gln synthesis via hepatic glutamine synthetase activity. Subsequently, Glu and Gln are important substrates for the synthesis of Asp from oxaloacetate by aspartate aminotransferase enzyme and Asn from Asp by asparagine synthetase, respectively [48]. These biochemical conversions may plausibly explain the selective increase in precursor amino acids for the urea cycle (Glu, Gln, Asp) despite the fact that plasma urea concentration was not measurably altered. In a recent study with dairy cows, a similar source of menthol-rich PBLC even decreased blood and milk urea levels whereas milk protein yield increased [46]. The latter suggests that increased synthesis of ureagenic amino acids may potentially contribute to improved protein efficiency.

There is relatively little information regarding the effects of menthol or other PBLC on the haematogram in ruminants. A PBLC mixture (ricinoleic and anacardic acid, cardanol and cardol) fed to bulls (0.42 g/kg DM) did not change any haemato-biochemical variables [49]. In rams, feeding of *Mentha piperita-*treated silage (20 mL/kg diet) increased WBC count, but had no effect on RBC numbers [50]. In the present study, haematological values were within the reported reference ranges for sheep [51, 52] and were not affected by treatment except for neutrophil count, which decreased linearly with increasing PBLC dose. We are not aware of any previous study showing an effect of menthol or other PBLC on blood neutrophil counts. However, few studies reported that certain PBLC suppressed neutrophil migration and accumulation in inflammatory sites [53–55]. Moreover, an oral administration of menthol reduced neutrophil counts in rats’ stomach suffering from ethanol-induced gastritis [56] and reduced the production of different inflammatory mediators [57, 58]. These observations support the view that the reduced neutrophil count in the present study may suggest a decreased inflammatory status and thus improved health resilience of sheep supplemented with menthol-rich PBLC.

One last intention of the present study was to investigate the effects of PBLC on the function of ruminal and jejunal epithelia with special focus on nutrient uptake. The results first confirmed the general textbook knowledge that the small intestine is the main site of glucose and amino acid absorption in ruminants. In the jejunum, uptakes of glucose and methionine were clearly dependent on the presence of Na^+^, which implies that Na^+^-dependent transporters have a significant contribution to the transport of these nutrients in the small intestine. Uptake of methionine from the rumen was negligible and not clearly Na^+^-dependent, which suggest that rumen has no quantitative significance for the absorption of methionine and likely also other amino acid. Ruminal glucose uptake tended to be greater in the presence versus the absence of Na^+^. This confirmed results of previous studies demonstrating Na^+^-dependent absorption of glucose from the rumen [26,27,59]. Furthermore, the present study identified, for the first time, a positive correlation between blood glucose concentration and ruminal glucose uptake. This is remarkable and supports the functional relevance of a previously postulated sugar-sensing mechanism on the basolateral side of ruminal epithelia. An earlier study had shown that serosal pre-incubation with *D*-glucose, *D*-mannose, 3-O-methyl-*D*-glucose or sucrose can up-regulate apical glucose uptake of isolated ruminal epithelia via such sensor [60]. It was speculated in that previous publication that the functional relevance of this mechanism is to adapt ruminal glucose absorption to the supply of easily fermentable carbohydrates, especially sugars, to the rumen because increased sugar supply to the rumen can effectively trigger an increase in blood glucose [60]. This is different from the intestine of sheep where upregulation of glucose absorption is also triggered by an increased supply of sugars but the sensor is luminal [61–63].

Despite the absence of data on menthol, we had speculated about an influence of menthol-rich PBLC on apical glucose or methionine uptake based on previous publications showing either stimulatory (thymol and carvacrol mixture [64] or cinnamaldehyde [14]) or inhibitory effects (*trans*-resveratrol and ε-viniferin [19] or quercetin-3-O-glucoside [18]) of certain other secondary plant metabolites on nutrient uptake. Our results showed that apical glucose and methionine uptakes in ruminal epithelia were not influenced by PBLC under Na^+^-containing conditions, which suggests that Na^+^-dependent glucose cotransporters (e.g., SGLT1 [26, 27, 65]) or Na^+^-dependent methionine transporters (e.g., B^0^AT1, ATB^0^+, IMINO and ASCT2 [66]) were not affected by pre-feeding menthol-rich PBLC. However, Na^+^-independent ruminal uptakes of both glucose and methionine increased in PBLC pre-fed sheep at the higher dose (160 mg/d). Increased uptakes of glucose and methionine from the ruminal epithelia in the absence of Na^+^ may involve Na^+^-independent transport facilitators (e.g., GLUT-2 and GLUT-5 for glucose, and b^0^AT for methionine [27,65–67]), which could potentially be stimulated by PBLC. Alternatively, this may indicate an upregulation of a more general unspecific pathway of passive substrate uptake like the paracellular route.

Another indication for an opening of the paracellular space in the rumen may be taken from the increase of *G*_t_ following supplementation with menthol-rich PBLC, which was restricted to the rumen but not the jejunum. The paracellular space is an unspecific pathway for passive ion permeation and thus contributes significantly to *G*_t_ [68**]**. The paracellular space is much tighter in the rumen than the jejunum [68**]**; thus even small increases in paracellular permeability are readily accompanied by increases in *G*_t_ [69]. Alternatively, increases in *G*_t_ may also point to an opening of ion channel conductivities, especially, if they concur with increases in *I*_sc_. An increase of *I*_sc_ was observed in the rumen and, as a trend, in the small intestine of the PBLC-supplemented sheep and reflects either enforced transcellular cation absorption or enforced transcellular anion secretion. Menthol is a potent activator of TRPM8 and TRPV3, the latter being involved in cation absorption across gastrointestinal epithelia. The ruminal epithelium contains several TRP channels, including TRPV3. The addition of menthol to isolated ruminal epithelium *ex vivo* has been shown to lead to parallel increases of *I*_sc_ and *G*_t_, which were electrophysiological correlates of increased absorptive fluxes of cations like calcium and ammonium in previous studies [16,17].

Viewing all findings of the present study synoptically, it appears that addition of menthol-rich PBLC in the tested dose range offers several beneficial effects. The majority of findings were based on linear effects or trends for linear effects with a more prominent appearance in PBLC-H. This indicates that increasing benefits are possible with increasing doses up to a total dose of 114 mg menthol per kg DMI. The latter appears to be a rather low dose compared to many literature studies. When assessing the dose holistically, however, safety has to be considered as well. In the EU, for example, menthol supplementation is judged unrestrictedly safe up to a concentration of 25 mg/kg complete feeding stuff. Exceeding this concentration is possible but requires detailed labelling [70].

## Conclusions

The present study revealed a potential for use of menthol-rich PBLC in growing sheep in doses up to ~ 100 mg/kg DMI. The applied PBLC improved feed intake but had no measurable short-term effect on growth. Linear decreases in neutrophil count and a trend for linear increases in serum calcium levels after PBLC addition could point to attenuation of inflammatory reactions and positive effects on calcium homeostasis, respectively, when considering other literature findings. An activation of TRP channels in the gastrointestinal tract by menthol could jointly explain the observed trend for higher serum calcium levels (via increased calcium absorption) and the selective linear increase in urea precursor amino acids in blood serum of PBLC-supplemented sheep (via increased ruminal absorption of NH_4_^+^). Further analysis of ruminal and jejunal functions *ex vivo* revealed increased Na^+^-independent uptakes of glucose and methionine and increased ion currents and permeability in the rumen especially at the higher dose of PBLC supplementation. These altered epithelial functions may indicate either an opening of a common unspecific transport pathway, e.g. the paracellular space, and/or coordinated upregulation of several selective transport and permeation pathways, again, including TRP channels.

## Data Availability

All data that support the findings of this study are included in this published article.

## Abbreviations

ADF_om_: Acid detergent fibre expressed exclusive of ash
CP: Crude protein
DM: Dry matter
DMI: Dry matter intake
EE: Ether extract
*G*_t_: Tissue conductance
*I*_sc_: Short-circuit current
MCH: Mean corpuscular haemoglobin
MCHC: Mean corpuscular haemoglobin concentration
MCV: Mean corpuscular volume
MPV: Mean platelet volume
NDF_om_: Neutral detergent fibre expressed exclusive of ash
OM: Organic matter
PBLC: Plant bioactive lipid compounds
PBLC-H: PBLC at a higher dose (160 mg/d)
PBLC-L: PBLC at a lower dose (80 mg/d)
PCT: Plateletcrit
PCV: Packed cell volume
*PD*_t_: Potential difference
PDWc: Platelet distribution width (co-efficient of variations)
PDWs: Platelet distribution width (standard deviation)
RDWc: Red cell distribution width (co-efficient of variations)
RDWs: Red cell distribution width (standard deviation)
SCFA: Short-chain fatty acid
TRP: Transient receptor potential
